# Editorial: Eating disorders and emotion regulation, looking at the spectrum from overcontrolling to dysregulation

**DOI:** 10.3389/fpsyt.2022.976500

**Published:** 2022-07-18

**Authors:** Paolo Meneguzzo, Anna Victoria Oldershaw, Francesco Monaco, Katrin Giel

**Affiliations:** ^1^Department of Neuroscience, University of Padova, Padova, Italy; ^2^Padova Neuroscience Center, University of Padova, Padova, Italy; ^3^Kent and Medway All Age Eating Disorder Service, North East London NHS Foundation Trust, Kent, United Kingdom; ^4^Salomons Institute for Applied Psychology, Canterbury Christ Church University, Royal Tunbridge Wells, United Kingdom; ^5^Department of Mental Health, ASL Salerno, Salerno, Italy; ^6^Department of Psychosomatic Medicine and Psychotherapy, Medical University Hospital Tübingen, Tübingen, Germany

**Keywords:** eating disorders, emotion regulation, overcontrol, dysregulation, emotional spectrum

Emotion regulation (ER) is defined as the ability to exert control over one's own emotional state. In recent years, extensive research has found ER to be a transdiagnostic risk factor involved in eating disorders (1). People with anorexia nervosa present associated cognitive-affective models that play a role in the management of maladaptive schemata (2), with no differences between individuals with anorexia nervosa or bulimia nervosa (3). Finally, in binge eating disorder, a distinct neurobiological phenotype has been proposed, integrating emotion regulation and impulsivity as the core of the development and maintenance of the disorder (4). Different treatment approaches have been evaluated across the literature, showing the potential role of specific cognitive training (5), specific treatment approaches (6), or integration in rehabilitation protocols (7). However, the basis of the vulnerability to use maladaptive ED strategies – like rumination, suppression, or avoidance of emotions – has not been totally identified yet, calling for more investigation in this specific psychopathological element.

The articles included in this Topic, which present a wide range of research designs and methodologies and theoretical frameworks, reflect the extent of the literature that is being conducted on ER and related processes in eating disorders.

Leppaned et al. performed a network meta-analysis to explore associations between maladaptive ER strategies and eating psychopathology. They found that ruminations and non-acceptance of emotions were most closely associated with specific eating psychopathology. They discussed the role of specific interventions, including in patients with low BMI that showed weaker associations.

Beeler and Burghardt proposed a neurobiological model for individuals with anorexia nervosa by looking at the possible role of dopamine in the continuums of the disorder, from the early stages to the maintaining behaviors. The model proposed should be evaluated and implemented in future studies, given the implications that it might have in treating approaches.

Drinkwater et al. performed a qualitative study on the experience of anorexia, emotions, and their management, supporting the emotion-focused models of anorexia. Indeed, they identified three distinct but interrelated phases during the recovery pathway: (1) coping with uncertainty, (2) seeing through the façade, and (3) growth. This study could help the future development of interventions, which might help individuals to recover.

Mikhail critically reviewed the studies that examined the associations between negative affect and loss of control eating, proposing an expanded affect-focused model that embraced trait-level individual differences, as well as biological and environmental variables.

Vasiliu performed a review of the existing literature on food addiction, showing the possible role of ER in this disorder, as well as the potential therapeutic solutions available now. Looking at the transdiagnostic role of ER in eating disorders, obesity, and food addiction, future studies should aim to implement strategies for the benefit of patients.

Li et al. showed the burden of eating disorders in China in the last decades, pointing out unexpected data about gender and dysfunctional behavioral differences that the previous literature has underestimated.

The current Research Topic generated a diverse collection of studies on ER and eating disorders. The collection includes different methodologies and populations, corroborating the transdiagnostic role of ER and its potential role in improving our understanding of psychopathology.

## Author contributions

PM wrote and prepare the first draft of the manuscript. AO, FM, and KG revised and supervised the first draft. All authors have approved the submitted version.

## Conflict of interest

The authors declare that the research was conducted in the absence of any commercial or financial relationships that could be construed as a potential conflict of interest.

## Publisher's note

All claims expressed in this article are solely those of the authors and do not necessarily represent those of their affiliated organizations, or those of the publisher, the editors and the reviewers. Any product that may be evaluated in this article, or claim that may be made by its manufacturer, is not guaranteed or endorsed by the publisher.
